# α‐Glucosidase Inhibitory Potential of *Citrus reticulata* Peel‐Derived Flavonoids—A Prelude for the Management of Type 2 Diabetes

**DOI:** 10.1002/fsn3.71499

**Published:** 2026-02-01

**Authors:** Itumeleng T. Baloyi, Ali H. Rabbad, Ntombenhle H. Gama, Samkelo Malgas

**Affiliations:** ^1^ Department of Biochemistry, Genetics & Microbiology University of Pretoria Hatfield South Africa

**Keywords:** α‐glucosidase inhibitors, citrus reticulata, flavonoids, type 2 diabetes

## Abstract

α‐Glucosidase inhibitors (AGIs) are compounds used to treat type 2 diabetes (T2D) by preventing the breakdown of dietary starch into monosaccharides, which reduces their absorption by the body and lowers blood glucose levels. AGIs often cause gastrointestinal issues such as diarrhea and flatulence due to excessive α‐amylase inhibition, leading to excess residual starch reaching the colon and being fermented by microbes. There is a need to prospect for novel AGIs that are effective and have fewer adverse effects. This study investigated the potential of citrus‐derived flavonoids as AGIs targeting amylolytic enzymes: α‐amylase and α‐glucosidase. Firstly, flavonoids were extracted from *Citrus reticulata* (tangerines) peels using an ultrasound‐assisted methanolic procedure, followed by C18 column‐purification and profiling with liquid chromatography‐mass spectrometry. Select citrus peel‐derived flavonoids, quercetin (−9.2 kcal/mol) and rutin (−10.8 kcal/mol), and the commercial AGI, acarbose (−8.7 kcal/mol), showed strong binding affinities against α‐glucosidase. Molecular dynamics simulations of the compounds were also assessed, revealing flexibility and stability in response to ligand interactions with the α‐glucosidase. The in silico data correlated positively with the results from the in vitro inhibition assays; acarbose (Ki = 0.14 mg/mL), quercetin (Ki = 0.12 mg/mL) and rutin (Ki = 0.19 mg/mL) recorded low inhibition constant values. The cytotoxicity profile of the selected compounds was also conducted on Caco‐2 cells, with flavonoids showing no significant cytotoxic effects. Flavonoids could be used as AGIs with minimal gastrointestinal impacts, reducing residual starch entering the colon and decreasing glucose uptake.

## Graphical abstract

Flavonoids derived from *Citrus reticulate* peel inhibit α‐glucosidase activity, delaying carbohydrate digestion and reducing glucose absorption, thereby indicating their potential as functional food components for type 2 diabetes management.

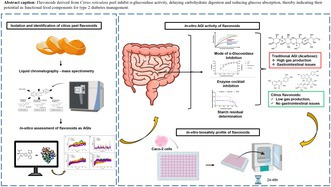

## 1 Introduction

Diabetes mellitus is a serious metabolic disease with a high incidence worldwide. Diabetes has emerged as the world's third “silent killer” following cancer and cardiovascular disease, due to its rising morbidity and fatality rates (Choudhury and Devi Rajeswari 2021). It is characterized by insufficient insulin production or resistance to insulin due to genetic and environmental factors, such as obesity, oxidative stress, and poor dietary choices (Kodikonda and Naik 2017). In the small intestine, the enzymes α‐glucosidase and α‐amylase play a crucial role in breaking down long‐chain carbohydrates into simple glucose molecules, facilitating their transport into the cells (Sekar et al. 2019). Inhibiting these carbohydrate‐degrading enzymes is one of the therapeutic ways to stabilize blood glucose levels and manage type 2 diabetes (T2D), and such therapeutics are called α‐glucosidase inhibitors (AGIs) (Ghauri et al. 2021).

Currently, several AGIs are in clinical use, such as acarbose; however, most of these have limited therapeutic efficacy due to associated side effects, including hypoglycemia and gastrointestinal issues (flatulence, abdominal distension, and diarrhea) (Wang et al. 2022). This is primarily due to the significant inhibitory effects against α‐amylase compared to α‐glucosidase. AGIs inhibit carbohydrate breakdown in the upper small intestine by binding to maltase‐glucoamylase and sucrase‐isomaltase, which delays and decreases glucose absorption into the blood (Takewaki et al. 2021). They are known for reducing post‐meal glucose spikes by inhibiting polysaccharide digestion. Consequently, a large portion of complex carbohydrates and starches remain undigested throughout the gastrointestinal tract, potentially altering the gut microbiome's structure and function, as microbial species there convert various carbohydrates into simple sugars for energy (Lam et al. 2024). Acarbose raises the levels of starch that reach the colon, promoting carbohydrate‐degrading microbes and their fermentation products (Takewaki et al. 2021). It has been demonstrated to increase the relative amounts of Bifidobacterium and Lactobacillus in the gut microbiota of T2DM patients, while reducing Bacteroides, significantly affecting the gut ecosystem (Zhong et al. 2025). Bacterial species in the colon ultimately break down and ferment these residual carbohydrates, producing gas that causes side effects such as bloating. Therefore, when prospecting for novel AGIs, it is important to prioritize compounds with strong α‐glucosidase inhibition, with mild to no α‐amylase inhibition (Lam et al. 2024).

Citrus fruit processing produces about 15 million tons of by‐products annually, highlighting the importance of utilizing citrus waste for generating value‐added products that may be used in various sectors, such as nutraceutical applications (Munir et al. 2024). Citrus peels are rich in various flavonoids like hesperidin, naringin, and nobiletin, which are becoming a promising ingredient for promoting health (Munir et al. 2024). Flavonoids are (poly)phenolic compounds characterized by a C6–C3–C6 structure, consisting of two benzene rings (A and B rings) connected by a three‐carbon bridge. Typically, this three‐carbon structure creates a heterocyclic pyran ring (C ring) (Lam et al. 2024).

Numerous studies have underscored the potential of flavonoids in combating diabetes due to their significant inhibition of α‐glucosidase and moderate inhibition of α‐amylase (Proença et al. 2022). This makes them promising candidates for developing anti‐diabetic medications with minimal gastrointestinal side effects. However, most studies have relied on synthetic substrates like ρ‐nitrophenyl‐glucopyranoside (ρNPG) and assessed enzyme activity separately, which presents limitations. Chromogenic substrates such as pNPG and CNPG3 are synthetic and may not resemble the enzyme's natural substrates in terms of structure and binding, affecting observed enzyme specificity and affinity (Perry et al. 2007). For instance, ρNPG does not fully mimic natural carbohydrates such as maltose and sucrose, meaning that compounds effective against it may not inhibit enzymes acting on complex carbohydrates in vivo. Enzyme activity measured with chromogenic substrates frequently yields different kinetic parameters than those measured with oligosaccharides or polysaccharides, sometimes leading to overestimation of catalytic rates and inhibitor potency (Visvanathan et al. 2020). Substrate accessibility and enzyme‐substrate interactions can vary: short‐chain chromogenic substrates are often hydrolysed more readily, while longer oligosaccharides may exhibit different substrate preferences or catalytic properties (Zulfiqar et al. 2023). Because chromogenic substrates engage only a limited number of subsites in the active‐site cleft, they do not reproduce the extensive hydrogen‐bonding networks formed by natural polysaccharides. As a result, transition‐state stabilization is altered and subsite cooperativity is reduced (Zulfiqar et al. 2023). Additionally, separate testing of α‐amylase and α‐glucosidase overlooks potential synergistic effects during carbohydrate digestion, leading to incomplete predictions of their therapeutic efficacy.

This study aimed to explore the in silico and in vitro inhibition of α‐glucosidase by citrus‐derived flavonoids as potential AGIs with fewer side effects. Maltose was utilized as the substrate for evaluating α‐glucosidase inhibition, instead of ρNPG. The effectiveness of the flavonoids against an amylolytic cocktail, comprised of both α‐amylase and α‐glucosidase, in mimicking carbohydrate digestion, was assessed. This method seeks to provide a more accurate prediction of the therapeutic efficacy of flavonoids. Additionally, we examined the residual starch content and size after inhibiting the enzyme cocktail to determine which inhibitors allowed for lower starch transport to the colon. To the best of our knowledge, this is the first study documenting the inhibition potential of flavonoids against α‐amylase and α‐glucosidase in this manner.

## 2 Materials and Methods

### 2.1 Materials

The following reagents and compounds were purchased from Sigma‐Aldrich Co. (St. Louis, MO, USA): Acarbose, cisplatin, diosmin, 3,5‐dinitro salicylic acid (DNSA), hesperidin, hog pancreatic α‐amylase, maltose monohydrate, naringin, nobiletin, potato starch, quercetin hydrate, and rutin hydrate. The D‐glucose assay kit (glucose oxidase/peroxidase; GOPOD) and α‐glucosidase from *Saccharomyces cerevisiae* were purchased from Megazyme (Bray, LEN, Ireland).

### 2.2 Citrus Peel Collection, Preparation and Ultrasound‐Assisted Extraction of Flavonoids

Fresh, ripe *Citrus reticulata* (tangerines) were bought from a local market in Pretoria, South Africa. The fruit was peeled, and the peels were cleaned, oven‐dried, and ground into a fine powder with a Waring grinder (WSG30) (Stamford, CT, USA). A 10 g sample of the pulverized peels was extracted in 300 mL of methanol using an ultrasonic bath (9 L digital ultrasonic cleaner, Eins Sci, Johannesburg, South Africa) for 60 min at 60°C, with a frequency of 60 kHz and a power level of 120 W. After the ultrasonic‐assisted methanolic treatment, the extracts were allowed to cool and filtered through a Whatman no. 1 filter paper. The resulting solution was centrifuged at 4000 *g* for 4 min at 4°C. The supernatant was collected and mixed with two volumes of 95% ethanol to precipitate any remaining residues overnight. The extract was then concentrated using a rotary evaporator at 45°C. The samples were allowed to cool in a desiccator. Subsequently, 350 mg of the dried crude extract was dissolved in 10 mL of methanol and purified using a gravity‐flow C18 column. The eluted flow‐through and fractions were collected and then concentrated with a rotary evaporator set to 45°C. The yields of flavonoid extraction and fractionation were determined using Equation (1).

(1) $$\text{Yield}\;\left(\%\right)=\frac{\text{mass of flavonoid extract}}{\text{mass of citrus peel powder}}\times 100$$

### 2.3 Chemical Profiling of *C. reticulata* Peel‐Derived Flavonoids

Ultra‐performance liquid chromatography coupled with quadrupole time‐of‐flight mass spectrometry (UPLC‐UV‐qTOF/MS) was utilized for the tentative identification of flavonoids in the *C. reticulata* extract, following the methods of He et al. (2019) with some modifications. The sample was loaded onto an XBRIDGE UPLC C18 column (2.1 × 150 mm, 1.8 μm) from Waters Inc. (Milford, MA, USA), using a Waters Acquity Sample Manager. The eluted components were analyzed with an in‐line Acquity eλ PDA and a Xevo G2 qTOF detector. Compound separation was accomplished using a linear gradient elution method with mobile phases consisting of H2O (0.1% formic acid) as solvent A and MeOH (0.1% formic acid) as solvent B. The solvent program proceeded as follows: It began with 60% solvent B and was held for 1 min. This was followed by a gradual increase to 100% solvent B for 14 min. After reaching 100% solvent B, a 3‐min column wash was performed from 14 to 17 min. The system was returned to the initial conditions to equilibrate the column from 17.5 to 20 min. A constant column temperature of 50°C was maintained, with a uniform flow rate of 0.3 mL/min and an injection volume of 5.00 μL. Detection of the eluting compounds was performed only in positive electrospray ionization mode (ESI+).

### 2.4 In Silico Screening of Pharmacokinetic Properties of Flavonoids

The pharmacokinetic properties of the identified flavonoids were evaluated using the SwissADME web server (http://www.swissadme.ch/index.php) (Daina et al. 2017). The SMILES strings of the flavonoids were obtained from the PubChem database (https://pubchem.ncbi.nlm.nih.gov/) and submitted to the SwissADME web server for analysis to predict various parameters. The determination of pharmacokinetic profiles for each compound, with benchmarks set against acarbose.

### 2.5 Molecular Docking Screening of Flavonoids Against Amylolytic Enzymes

Molecular docking was conducted following the protocol established by Tshiyoyo et al. (2025) in our research laboratory, with minor modifications. The selected compounds were virtual screened into the active sites of two amylolytic enzymes using AutoDock Vina 1.1.2 within the PyRx 08 software (Morris et al. 2009; Dallakyan and Olson 2015). Enzyme preparation and visualization of ligand–protein interactions were done with Discovery Studio Visualizer v21.1.0 (San Diego, CA, USA). The 3D crystal structure of porcine pancreatic α‐amylase (PDB ID: 1DHK) was sourced from the Protein Data Bank (PDB). Due to the unavailability of the 3D structure of *Saccharomyces cerevisiae* α‐glucosidase, a model was generated using the Swiss‐MODEL web server. Both enzyme structures were refined by removing water molecules before docking. Ligand 3D structures were retrieved from PubChem, converted to PDB format, and imported into PyRx for energy minimization and docking. Docking parameters for α‐amylase included center coordinates of 102 × 40 × 18 Å and dimensions of 27 × 27 × 27 Å, while α‐glucosidase used center coordinates of 2.9 × 4.9 × −0.13 Å with the same dimensions. Post‐docking, ligand–enzyme complexes were visualized in Discovery Studio to create 2D interaction maps, showcasing key interactions in the active sites.

### 2.6 Inhibition of α‐Amylase by Citrus Peel Flavonoids

The α‐amylase inhibitory activity was evaluated following the method described by Karakaya et al. (2018) and Tshiyoyo et al. (2025), with slight modifications. The inhibition profile was assessed using acarbose (0–0.50 mg/mL) in combination with citrus peel extract and citrus‐derived mono‐component flavonoids—diosmin, hesperidin, naringin, nobiletin, quercetin, and rutin (0–2.00 mg/mL). The percentage of α‐amylase inhibition caused by these compounds was determined using Equation (2).

(2) $$\%\text{Inhibition}=\frac{A_{\text{control}-}A_{\text{sample}}}{A_{\text{control}}}\times 100$$

### 2.7 Inhibition of α‐Glucosidase by Flavonoids

The inhibition profile of α‐glucosidase was determined using acarbose (0–0.5 mg/mL), along with citrus extract and citrus‐derived flavonoids (diosmin, hesperidin, naringin, nobiletin, quercetin, and rutin) (0–2.00 mg/mL) as described previously by Tshiyoyo et al. (2025). The percentage of α‐glucosidase inhibition was calculated and determined as described in Section 2.6.

### 2.8 α‐Glucosidase Inhibition Kinetics by Flavonoids

In a 96‐well microplate, 100 μL of maltose solution at different concentrations (0%–2%) was mixed with 50 μL of flavonoids solution (0.5 or 1 mg/mL), or acarbose (0.125 or 0.250 mg/mL). After pre‐incubation, the reaction commenced with the addition of 50 μL of α‐glucosidase (40 μg/mL; 100 U/mg), followed by incubation at 37°C for 20 min. The amount of glucose produced was determined as outlined in Section 2.7. Lineweaver‐Burk (LB) plots displaying 1/V versus 1/[substrate] were constructed using GraphPad Prism to determine the Michaelis–Menten constant (*K*_m_) and the maximum velocity (*V*_max_).

### 2.9 Molecular Dynamics Simulations of Potent α‐Glucosidase Inhibitory Flavonoids

Molecular dynamics (MD) simulations were performed to further explore the interactions between selected compounds and α‐glucosidase, confirming the docking study results. Before the MD simulations, UCSF Chimera was used to prepare the systems by adding hydrogen atoms and assigning AM1‐BCC charges. The MD simulations were carried out using the AMBER 18 Particle Mesh Ewald Molecular Dynamics (PMEMD) package on a single GPU. Atomic partial charges for all compounds were generated with the ANTECHAMBER module. Trajectories of 100 ns were collected at 1 ps intervals for the apo form of α‐glucosidase and for all other complexes. After simulations, the trajectories were analyzed with the CPTRAJ and PTRAJ modules of AMBER 18. RMSD analyses assessed the system's stability and rigidity, while RMSF evaluated the fluctuations of individual residues. The radius of gyration (RoG) and solvent‐accessible surface area (SASA) measurements provided insights into the system's compactness and solvent exposure. Finally, the MM/GBSA method was used to estimate the binding free energy of each compound (Rabbad et al. 2019; Tshiyoyo et al. 2025).

### 2.10 Inhibition of an Amylolytic Enzyme Cocktail by Flavonoids

The method for inhibiting the amylolytic enzymes cocktail followed the procedures outlined in Sections 2.6 and 2.7 with minor modifications. This approach aimed to inhibit the amylolytic enzyme cocktail, which includes α‐amylase and α‐glucosidase, using individual compounds: acarbose, quercetin, rutin, and nobiletin. Two hundred microlitres of 2% potato starch were incubated with 100 μL of varying acarbose, nobiletin, quercetin, and rutin in a 1.5 mL tube. The mixture was incubated for 5 min at 37°C on a Roto‐Therm Plus at 25 rpm. To start the reaction, 50 μL of 50 μg/mL hog pancreatic α‐amylase and 50 μL of 80 μg/mL *S. cerevisiae* α‐glucosidase were added, then incubated for 20 min at 37°C on the rotator. Enzymes were inactivated by 3 min at 100°C.

The inhibitory activities of the selected compounds were determined by profiling the hydrolysates using the (1) DNS method for total reducing sugars (TRS), (2) GOPOD method for glucose, (3) starch‐iodine method for residual starch content, and (4) dynamic light scattering for starch residue size distribution as described in our previous work by Tshiyoyo et al. (2025).

### 2.11 Evaluation of the Biocompatibility Profile of Flavonoids Using CaCO‐2 Cells

Selected potent α‐glucosidase inhibitory citrus flavonoids, quercetin and rutin, alongside acarbose, were evaluated for their cytotoxic effects on CaCO‐2 cells following a method described previously (Esghaei et al. 2018; Khuzwayo et al. 2025), with slight modifications. CaCO‐2 cells were cultured in Dulbecco's Modified Eagle Medium (DMEM) supplemented with the following components: 3.7 g/L sodium bicarbonate, 1.1% (v/v) of 100 mM sodium pyruvate, 2.5% (v/v) of 1 M HEPES, 0.05% (v/v) gentamicin (at a concentration of 10 mg/mL), 1% (v/v) of a 100× antibiotic‐antimycotic solution, and 10% (v/v) heat‐inactivated fetal bovine serum (FBS). When confluent, cells were trypsinised with 0.25% (v/v) trypsin–EDTA. Succinctly, 24 h before experiments, trypan blue was used for cell counting using the Bürker chamber to determine the number of cells (1 × 10^5^ cells/mL) to be seeded on the 96‐well microplates. The cells were then incubated overnight at 37°C in an atmosphere containing 5% (v/v) CO_2_ to promote optimal growth and adherence. Following this, a two‐fold dilution series of 100 μL test compounds and cisplatin (positive control) was prepared, ranging from 0 to 1 mg/mL. These dilutions were incubated with the cells for 48 h at 37°C and 5% (v/v) CO_2_. After incubation, the plates were centrifuged at 1200 rpm for 5 min, and 180 μL of media was aspirated and washed twice with an equivalent amount of phosphate‐buffered saline. The supernatant was aspirated, and 100 μL working MTT solution (5 mg/mL MTT in serum‐free DMEM media, in a 1:9 ratio) was added to each well in the dark and incubated for 4–24 h at 37°C and 5% (v/v) CO_2_. Formazan crystals were solubilized with 100 μL of solubilization solution (1 M HCl and isopropanol, in a 1:9 ratio). Plates were incubated in the dark for 15 min at room temperature. Absorbance was then measured at 540 and 690 nm (blank controls) using a SpectraMax Paradigm multi‐mode microplate reader (San Jose, California, USA). The cytotoxic effects and the selectivity index (SI) of the potent flavonoids were calculated using Equations (3) and (4), respectively.

(3) $$\text{Cell viability}\;\left(\%\right)=\frac{\mathrm{Abs}\;\left(540\;\mathrm{nm}\right)\;\text{treatment}-\mathrm{Abs}\;\left(690\;\mathrm{nm}\right)\;\text{blank}}{\mathrm{Abs}\;\left(540\;\mathrm{nm}\right)\;\text{untreated}-\mathrm{Abs}\;\left(690\;\mathrm{nm}\right)\text{blank}}\times 100$$

(4) $$\text{Selectivity index}=\frac{\text{Cytotoxicity concentration}\;\left({\mathrm{CC}}_{50}\right)}{\text{Inhibitory concentration}\;\left({\mathrm{IC}}_{50}\right)}$$

### 2.12 Statistical Analysis

All experiments were performed in triplicate with results expressed as mean ± SEM. Initial data analysis was performed using Excel on Windows 10, and further analysis was done with GraphPad Prism version 8 (San Diego, CA, USA). A one‐way ANOVA assessed treatment differences, with flavonoids as the grouping variable and concentration as the independent variable. Statistical significance was set at *p* < 0.05.

## 3 Results

### 3.1 Chemical Composition of *C. reticulata* Peels

The extraction yield of *C. reticulata* peels utilizing ultrasonic‐assisted extraction (UAE) with methanol was determined to be 26.48% (w/w). The yield for the flow through and fraction obtained was 20.58% and 4.96%, respectively. The flavonoids extracted from the peels were profiled using UPLC‐UV‐qTOF/MS, leading to the tentative identification of thirteen compounds through the UNIFI software platform (Figure S1, Table 1). Among these, notable compounds include etrogol, diosmin, and nobiletin; additionally, flavanone glycosides such as hesperidin, neohesperidin, and tangeritin were identified. Furthermore, polymethoxylated flavones, including nobiletin, hexamethylquercetagetin, and sinensetin, were also identified.

**TABLE 1:** Chemical composition of flavonoids of *C. reticulata* peels.

Retention time (min)	Component name	Mass fragment (m/z)	Chemical formula
5.82	Etrogol	207.1398	C_13_H_18_O_2_
7.26	Hesperetin 7‐O‐glucoside	465.1396	C_22_H_24_O_11_
7.27	Hesperidin^a^	611.1971	C_28_H_34_O_15_
7.47	Diosmin	609.1815	C_28_H_32_O_15_
9.77	Sinensetin	373.1295	C_20_H_20_O_7_
10.01	Isosinensetin	373.1295	C_20_H_20_O_7_
10.24	Gossypetin hexamethyl ether^b^	403.1395	C_21_H_22_O_8_
10.43	4′,5,6,7‐Tetramethoxyflavone	343.1189	C_19_H_18_O_6_
10.66	Nobiletin^b^	403.1395	C_21_H_22_O_8_
10.86	3‐Methoxynobiletin	433.1502	C_22_H_24_O_9_
10.95	Tangeretin	372.1209	C_20_H_20_O_7_
11.14	Demethylnobiletin	389.1243	C_20_H_20_O_8_
11.51	Hexamethylquercetagetin^b^	403.1399	C_21_H_22_O_8_

^a^ Isomer of neohesperidin.

^b^ Nobiletin and two of its isomers.

### 3.2 Pharmacokinetic Predictions of Acarbose and Identified Flavonoids

Acarbose, diosmin, hesperidin, and naringin exhibited lower bioavailability scores and intestinal absorption compared to nobiletin, quercetin, sinensetin, and tangeritin, which had higher bioavailability scores (> 0.5) (Table 2). Acarbose, diosmin, hesperidin, naringin, and rutin are shown not to be the CYP inhibitors but are Pgp substrates. All compounds, except for sinensetin and tangeritin, have shown an inability to cross the blood–brain barrier.

**TABLE 2:** Pharmacokinetic properties of acarbose and the identified *C. reticulata* flavonoids.

Compound	GI absorption	Bio‐availability	LogP	CYP inhibitor	Pgp substrate	BBB permeant
Acarbose	Low	0.17	−8.53	No	Yes	No
Diosmin	Low	0.17	−0.14	No	Yes	No
Hesperidin	Low	0.17	0.14	No	Yes	No
Naringin	Low	0.17	−0.14	No	Yes	No
Nobiletin	High	0.55	3.01	CYP2C9; CYP3A4	No	No
Quercetin	High	0.55	1.54	CYP1A2; CYP2D6; CYP3A4	No	No
Rutin	Low	0.55	−0.33	No	Yes	No
Sinensetin	High	0.55	3.50	CYP2C19; CYP2C9; CYP3A4	No	Yes
Tangeritin	High	0.55	3.04	CYP2C9; CYP3A4	No	Yes

### 3.3 Molecular Docking of Flavonoids Against Amylolytic Enzymes

In the case of α‐amylase, the compounds quercetin (−7.8 kcal/mol), rutin (−8.6 kcal/mol), hesperidin (−9.1 kcal/mol), and diosmin (−9.1 kcal/mol) exhibited better binding affinity compared to acarbose (−7.1 kcal/mol) as presented in Table 3. Additionally, these compounds demonstrated improved binding affinity on the active site of α‐glucosidase, with the following binding affinities: rutin (−10.8 kcal/mol), diosmin (−10.3 kcal/mol), hesperidin (−10.0 kcal/mol), quercetin (−9.2 kcal/mol), and acarbose (−8.7 kcal/mol). The interactions of the selected compounds with α‐glucosidase, including the protein residues involved, are presented in Figure S4. Overall, the compounds showed higher binding activity than acarbose for both α‐amylase (3AJ7) and α‐glucosidase.

**TABLE 3:** Binding affinity of citrus flavonoids against amylolytic enzymes, α‐amylase and α‐glucosidase.

Compound names	Binding affinity (kcal/mol)	
	α‐amylase	α‐glucosidase
Acarbose	−7.1	−8.7
Diosmin	−9.1	−10.3
Hesperidin	−9.1	−10.0
Naringin	−7.0	−7.8
Nobiletin	−6.4	−7.8
Quercetin	−7.8	−9.2
Rutin	−8.6	−10.8

### 3.4 The Inhibitory Effect and Mechanism of Action of Selected Flavonoids

The citrus peel‐derived flavonoid extract and the pure flavonoids did not show a strong inhibitory effect (IC_50_ > 2.00 mg/mL) against α‐amylase, while acarbose displayed a strongly inhibitory effect (IC_50_ = 0.011 ± 0.04 mg/mL). On the other hand, the crude and fractionated citrus peel extract showed weak inhibition, with IC_50_ values of 1.19 and 4.30 for α‐glucosidase. Two key selected flavonoids, quercetin and rutin, significantly inhibited α‐glucosidase with similar potency to acarbose (Table 4). The mode of inhibition of the compounds was competitive, and their Ki values were ordered as follows: acarbose > quercetin > rutin for α‐glucosidase inhibition (Table 4).

**TABLE 4:** Binding affinity, IC_50_, Ki, and mode of inhibition (MOI) of acarbose and potent citrus flavonoids against α‐glucosidase. Values are represented as means ±SEM (*n* = 3).

Compound name	Binding affinity (kcal/mol)	IC_50_ (mg/mL)	Ki (mg/mL)	Mode of inhibition
Acarbose	−8.7	0.19 ± 0.05	0.14 ± 0.02	Competitive
Quercetin	−9.2	0.42 ± 0.01	0.12 ± 0.01	Competitive
Rutin	−10.8	0.63 ± 0.06	0.19 ± 0.01	Competitive

### 3.5 MD Simulations of Selected Flavonoids‐α‐Glucosidase Interactions

Based on the in vitro inhibitory activity against α‐glucosidase, only selected flavonoids, quercetin and rutin, including acarbose as a standard inhibitor, were selected for the MD simulations to compare the stability and structural arrangement of the best‐docked ligand‐enzyme complexes with the unbound enzyme (apo enzyme) over 100 ns (Table S1).

The binding energy trend for α‐glucosidase interacting with acarbose and selected flavonoids exhibits more negative values, following acarbose, rutin, and quercetin. The two‐dimensional (2D) interactions between ligands and protein complexes were plotted using LigPlot to analyze the types of interactions formed after 100 ns for each compound (Figure S5). Acarbose has been shown to establish a hydrogen bond with Asp408, as illustrated in Figure S5A, and it is not accommodated within the α‐glucosidase binding site (Figure S5B).

**FIGURE 1:**
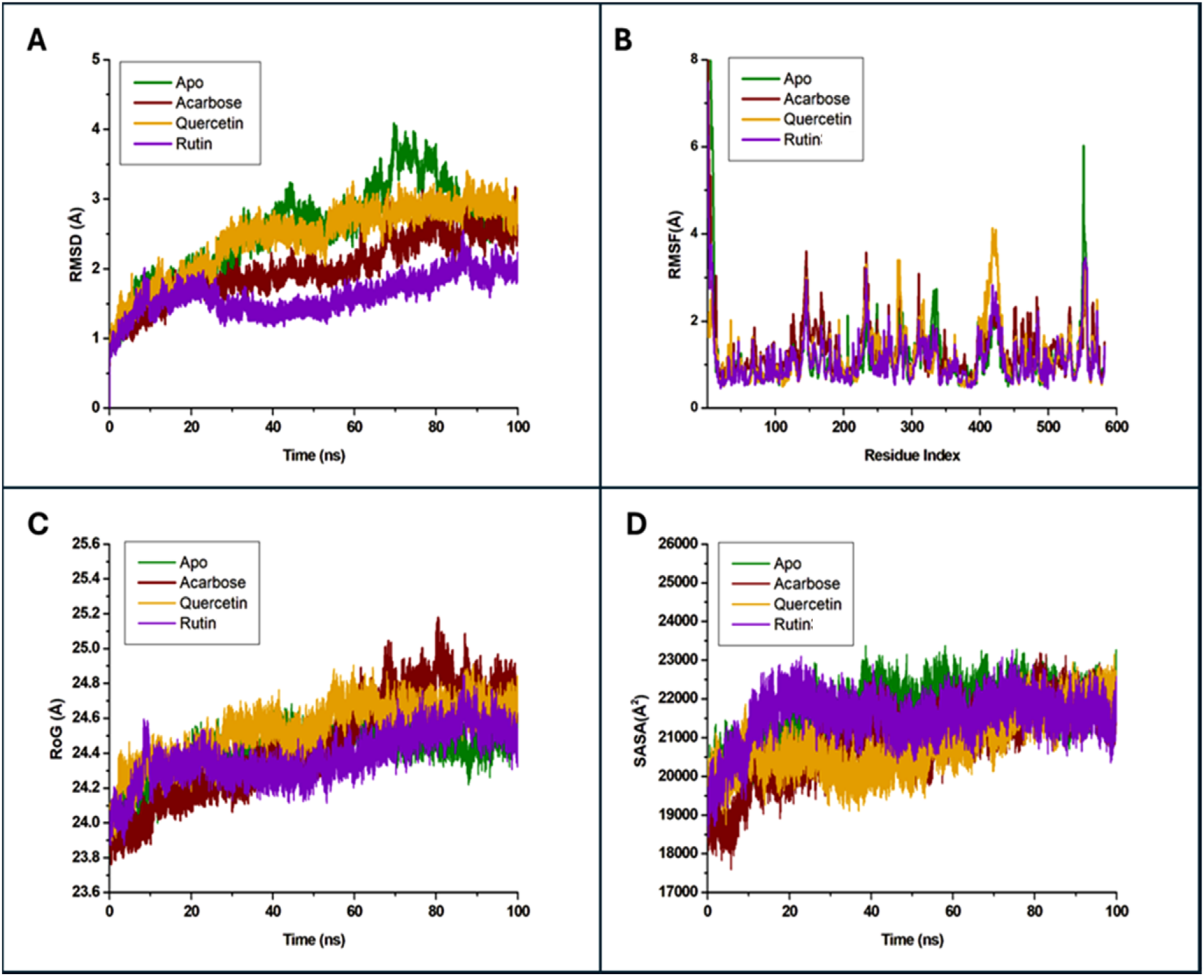
Molecular dynamics simulation of α‐glucosidase and its complexes with selected flavonoids for 100 ns. Analysis of (A) root mean deviation (RMSD); (B) root mean square fluctuations (RMSF); (C) radius of gyration (RoG) and (D) solvent‐accessible surface area (SASA).

Conversely, acarbose does form hydrogen bonds with Ser308, Arg312, Lys155, Asp214, and Arg212 (Figure S5C), and it interacts within the binding pocket of α‐glucosidase (Figure S5D). These results demonstrate the correct ligand interactions of acarbose, in line with the findings of the previous study. Quercetin formed hydrogen bonds with Asp408 and Arg312 (Figure S5E), while rutin formed hydrogen bonds with Asp408, Gln350, Asp349, and His279 (Figure S5F), and showed good binding affinity. The RMSD of both the apo‐protein and protein‐ligand complexes was analyzed to understand the flexibility and stability of the complexes (Figure 1). The apo‐protein, acarbose, quercetin, and rutin complexes were shown to have a similar RMSD trend within the 20 ns simulation time. The apo‐protein and quercetin showed a higher RSMD value of 2.5 Å (Figure 1A) after 20 ns, whereas the acarbose and rutin complexes exhibited RMSD values lower than 2.5 Å throughout the 100 ns simulation, indicating the stable state of these complexes. Acarbose and rutin demonstrated low fluctuations, indicating increased flexibility as presented in Figure 1B. Acarbose was shown to interact with the residues of Asp349 and Arg439 (Figure S5C), whereas rutin interacted with Asp349, Asp214, and Arg439, as shown in Figure S5F. The quercetin‐protein complex exhibited greater fluctuations in amino acid residues in the region 400–420 compared to acarbose and rutin (Figure 1B). In Figure 1C and Table S1, the apo α‐glucosidase showed a higher mean RoG value compared to complexes of acarbose, quercetin, and rutin, indicating that the complexes did not fluctuate significantly, demonstrating a stable profile of the complexes. The apo α‐glucosidase has a higher mean SASA than its complex with acarbose, quercetin, and rutin, indicating a stronger binding affinity and a more stable, folded structure during interaction with these ligands (Figure 1D). Overall, acarbose, quercetin, and rutin demonstrated to be flexible and stable structures with α‐glucosidase within the 100 ns simulation time.

### 3.6 The Effect of Selected Flavonoids on Inhibiting an Amylolytic Enzyme Cocktail

**FIGURE 2:**
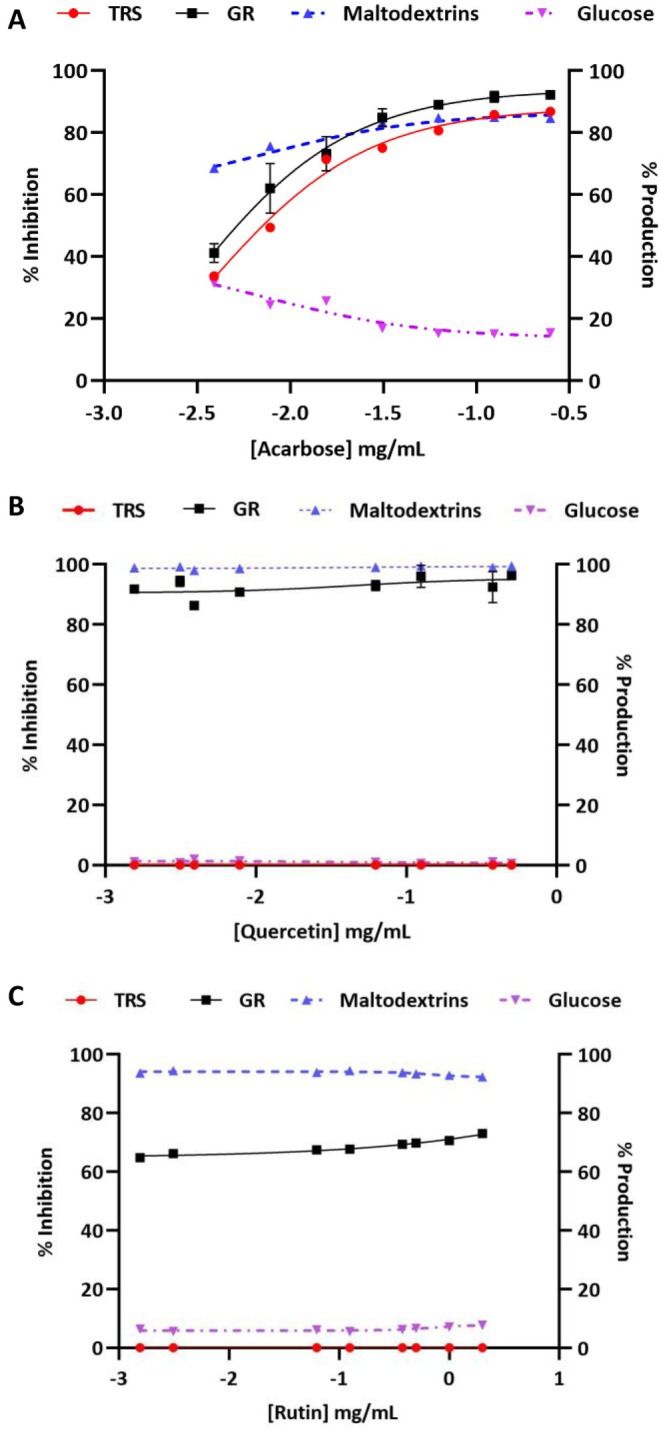
Amylolytic enzyme cocktail (α‐amylase and α‐glucosidase) inhibition by (A) acarbose, (B) quercetin and (C) rutin. The primary *y*‐axis corresponds to the % inhibition of total reducing sugars (TRS) and the glucose released (GR). The secondary *y*‐axis corresponds to the proportion of maltodextrins and glucose from TRS.

The potent flavonoids, quercetin and rutin, along with the standard inhibitor, acarbose, were evaluated for their ability to inhibit the amylolytic enzyme cocktail. Acarbose exhibited a significant inhibition of both α‐amylase and α‐glucosidase, as illustrated in Figure 2A.

In contrast, the flavonoids, quercetin and rutin, led to the production of a higher percentage of maltodextrins while significantly reducing glucose production. This suggests a more potent inhibition of α‐glucosidase compared to α‐amylase, as demonstrated in Figure 2B,C, respectively, which coincided with the IC_50_ results conducted on the two enzymes separately.

### 3.7 Determination of Starch Content and Particle Size in the Hydrolysates

**FIGURE 3:**
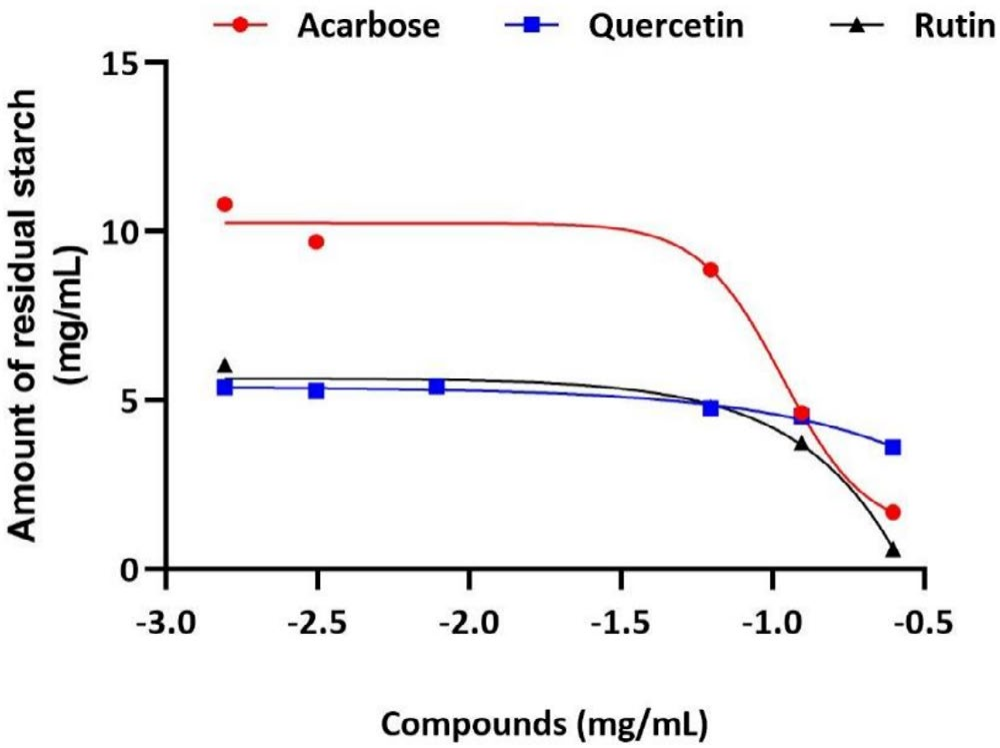
The residual starch amount after hydrolysis of 10 mg/mL starch was assessed in the presence of acarbose, quercetin, and/or rutin.

To confirm the undigested starch of acarbose, quercetin, and rutin inhibition of the amylolytic enzymes cocktail, the hydrolysates were analyzed for the particle size distribution of their starch content. The particle sizes of starch residues for amylolytic enzyme hydrolysates in the presence of acarbose, quercetin, and rutin were 140.05, 7.13, and 12.57 nm, respectively, compared to the starch control alone (156.24 nm) and starch hydrolysate without any inhibitor present (5.33 nm) (Table S2). In summary, there was a correlation of data between residual starch content in the hydrolysate (Figure 3) and particle size distribution of these starch residues.

### 3.8 Cytotoxic Effects of the Selected Flavonoids

The biocompatibility profile results indicated that both quercetin and rutin exhibited no cytotoxic effects on the Caco‐2 cells when tested in a dose‐dependent manner (Figure S6). The calculated cytotoxic concentration (CC_50_) values for quercetin and rutin were 1.54 and 6.15 mg/mL, respectively (Table 5). The selectivity index of acarbose, quercetin, and rutin was 2.58, 3.67, and 9.76, respectively. In contrast, acarbose and cisplatin demonstrated significant cytotoxicity, as evidenced by their detrimental effects on cell viability when concentrations surpassed critical thresholds of 0.49 mg/mL for acarbose and 0.57 mg/mL for cisplatin.

**TABLE 5:** Cytotoxic concentration (CC_50_) values for the assessed compounds on Caco‐2 cells, α‐glucosidase inhibitory concentration (IC_50_) values and selectivity indexes (SI) of potent compounds. Mean values are represented as ±SEM (*n* = 3).

Compound name	Caco‐2 cell (CC_50_) value (mg/mL)	α‐glucosidase IC_50_ value (mg/mL)	Selectivity index (SI)
Cisplatin	0.57 ± 0.03	n.d	n.d
Acarbose	0.49 ± 0.09	0.19 ± 0.05	2.58
Quercetin	1.54 ± 0.10	0.42 ± 0.01	3.67
Rutin	6.15 ± 0.10	0.63 ± 0.06	9.76

*Note:* Where (n.d) indicates not determined.

## 4 Discussion

Extraction is a critical and delicate step in recovering bioactive compounds from plant sources due to the sensitivity of the target molecules. This finding aligns closely with results reported by Yaqoob et al. (2023), who observed a flavonoid yield of 26.88% (w/w) from *C. reticulata* peels under similar extraction conditions. While different organic solvents are commonly employed for extracting phytochemicals, they frequently provide limited efficiency, require high temperatures, and yield relatively small amounts (Munir et al. 2024). It is important to note that the flavonoid profile of *C. reticulata* peels extends beyond these thirteen compounds, as additional flavonoids like quercetin, naritutin, and rutin have been documented (Yaqoob et al. 2023).

The pharmacokinetic properties of acarbose and the identified flavonoids were predicted using the SwissADME web server. Research into citrus flavonoids' absorption, distribution, metabolism, excretion and toxicity (ADMET) properties will enhance our understanding of their bio‐compatibility as phytochemicals (Wang et al. 2021). Flavanone glycosides are more hydrophilic than their aglycones and PMFs; hence, their solubility in water remains limited, particularly in the case of hesperidin and naringin (Zhang et al. 2021). Aglycone flavanones and polymethoxyflavones are more hydrophobic and can readily permeate membranes, with log *p* > 2 (Zhang et al. 2021). The identified polymethoxyflavones (PMFs), such as nobiletin, sinensetin, and tangeritin, demonstrated high permeability with log *p* > 2.

Most of the flavonoids are P‐gp (permeability glycoprotein) substrates, meaning they can be pumped back into the gut lumen, reducing their absorption. Lastly, flavonoids were reported not to be inhibitors of cytochrome P450 (CYP), suggesting that the flavonoids are less likely to cause metabolic drug interactions and cannot penetrate the blood–brain barrier (BBB).

Overall, the computational ADMET assessment suggests that flavonoids might pose minimal safety concerns when used by humans as phytochemicals.

Based on the results of the pharmacokinetic and ADMET properties, most flavonoids were screened for their interactions with the active sites of the amylolytic enzymes using porcine α‐amylase (1DHK) and α‐glucosidase from *S. cerevisiae* (MAL32). The SwissModel web server was employed to validate the α‐glucosidase model structure, focusing on the quality of the generated protein structure. The isomaltase (PDB ID: 3AJ7) was selected as the template due to its 72.51% similarity with the MAL32 *S. cerevisiae* sequence. This template was favored because it completely covered the query sequence (1.00) and boasted an excellent X‐ray crystallographic resolution of 1.3 Å, superior to other templates. Figure S2 illustrates the comparison of protein sequence similarity and identity between the model template and *S. cerevisiae* α‐glucosidase. Moreover, we present the 3D structure of the homology model developed for MAL32, along with its corresponding Ramachandran plot showing that 96.38% of the residues fall within the favored area, accompanied by a MolProbity score of 0.76 (Figure S3). A model exhibiting a high percentage of residues in the Ramachandran‐favored zones and a MolProbity score near or below 1 is deemed to have a high‐quality structure. A greater binding affinity for α‐glucosidase was observed among the compounds tested. The purpose of the in silico analysis is to comprehend and provide insight into how each compound interacts with the specific enzymes. Therefore, the pure, commercially sourced flavonoids, guided by the citrus peel flavonoid profile, and the extract were subsequently used to evaluate their in vitro inhibitory effect against the two amylolytic enzymes, α‐amylase and α‐glucosidase. Findings from this study indicate that the tested flavonoids inhibit α‐glucosidase more effectively than α‐amylase. Similarly, Shen et al. (2012) reported that four flavonoids (nobiletin, naringin, hesperidin, and neohesperidin) demonstrated only slight inhibitory effects on α‐glucosidase; the inhibition percentages were under 20%, even at the highest concentration (100 μM). A mild reduction of α‐amylase is typically favored to prevent excessive bacterial fermentation, which can cause gastrointestinal side effects, with the more desired effect being significant α‐glucosidase inhibition.

In the current study, the IC_50_ values were ranked as follows: acarbose > quercetin > rutin. A similar inhibition profile of acarbose > quercetin > rutin has been reported previously (Wang et al. 2025). Based on the effectiveness of these flavonoids, their enzyme kinetics studies were conducted to better understand how the compounds inhibit α‐glucosidase. The three tested compounds were shown to be competitive inhibitors of α‐glucosidase. Similar to this study, Zhang et al. (2017) also found acarbose to be a competitive inhibitor of α‐glucosidase.

It is stated that the Ki value can serve as an effective measure for comparing the inhibitory activity of phytochemical compounds, as it indicates how strongly an inhibitor binds to the protein. A smaller Ki value indicates a greater binding affinity (Tolmie et al. 2021). This has been proven in this study based on the correlation between the docking score and Ki value results (Table 4). A study conducted by our research team, Tshiyoyo et al. (2025), revealed comparable results regarding citrus essential oils, which demonstrated a strong correlation between Ki values and binding affinity scores generated from in silico results toward α‐glucosidase. Overall, the Ki values of the selected flavonoids have revealed a consistent in vitro measurement that illustrates their binding affinity for α‐glucosidase, highlighting the strength of their interactions with this enzyme. It is common practice to confirm the static model of the ligand‐protein interaction through MD simulations to evaluate the stability of the complexes and to calculate various MD trajectory parameters such as RMSD and RMSF (Ogunyemi et al. 2023). We discovered that the binding site identified in our earlier research (Tshiyoyo et al. 2025) was less accurate compared to the findings in this study. The inhibition of α‐glucosidase requires several active site residues to be involved, including Lys156, Glu276, Phe303, His351, and Arg312, as noted by Mehmood et al. (2022) and Rahim et al. (2015). Both acarbose and quercetin formed hydrogen bonds with the residue of Arg312, which could be attributed to the strong binding affinity of these compounds.

Pan et al. (2025) have reported similar fluctuations for a quercetin‐protein complex. RMSF values were measured to analyze the flexibility of the protein backbone for 100 ns. The active site of α‐glucosidase is located near the residues Asp349, Asp214, and Arg439 (Asadi et al. 2024).

RoG is frequently utilized as a characterization parameter to evaluate alterations in protein structure, to monitor protein integrity, and densification (Liu et al. 2023). The SASA of the α‐glucosidase complexes was evaluated to understand the differences in surface area (Swargiary et al. 2023). SASA helps comprehend the stability, molecular interactions, and function of proteins associated with protein folding, ligand binding, and conformational changes (Tshiyoyo et al. 2025).

The inhibition of carbohydrate‐hydrolysing enzymes such as α‐amylase and α‐glucosidase is crucial in the treatment/management of T2D (Oboh et al. 2015). A previous study showed that a combination of quercetin and rutin was potent in inhibiting α‐amylase and α‐glucosidase, with quercetin exhibiting a strong synergistic effect on α‐glucosidase inhibition (Oboh et al. 2015). This aligns with our research, which indicates that flavonoids display a weaker inhibitory effect against α‐amylase activity while demonstrating a more potent inhibition of α‐glucosidase activity. Consequently, these inhibitors could serve as effective treatments for T2D with minimal side effects resulting from colonic fermentation of residual starch (Kwon et al. 2006). The inhibition of the amylolytic enzyme cocktail by acarbose led to a notable portion of starch remaining undigested, as evidenced by the data presented in Figure 3. This finding corroborated the findings shown by the hydrolysate profile of the acarbose‐inhibited amylolytic enzyme cocktail (Figure 2A). On the other hand, quercetin and rutin revealed a reduced amount of residual starch; such outcomes are particularly desirable because they will have fewer gastrointestinal issues (Figure 3). This implies that the flavonoids attenuate glucose release by targeting α‐glucosidase and do not inhibit α‐amylase, further enhancing their effectiveness in carbohydrate digestion management.

Cytotoxicity assessment of the lead AGIs was conducted on Caco‐2 cells to predict their effect in the small intestine, an organ rich in carbohydrate‐hydrolysing enzymes (Tshiyoyo et al. 2022). To establish a comparative benchmark, cisplatin was employed as a positive control for cytotoxicity in these experiments. Compounds having a selectivity index value above 1 are desirable for effective AGI while minimizing cytotoxicity effects (Khuzwayo et al. 2025). These findings suggest that both compounds possess a favorable safety profile for a short‐term period, indicating that they could be safely considered for long‐term exposure, with additional research. The low cytotoxicity of the selected flavonoids underscores the importance of thoroughly understanding the cytotoxicity profiles of these agents to ensure cellular health and safety in future applications.

## 5 Conclusion

Flavonoids extracted from the peels of *C. reticulata* have shown promise as potential AGIs, with quercetin and rutin being notable for their strong inhibition of α‐glucosidase and lower inhibitory activity on α‐amylase. The use of maltose as a relevant substrate in assessing inhibition of the amylolytic enzyme and evaluating the residual starch in our study has highlighted a more realistic prediction of the therapeutic potential of the flavonoids and their capacity to limit starch passage to the colon. This indicates the potential for more effective treatments of type 2 diabetes (T2D), suggesting a reduced likelihood of digestive discomfort arising from the fermentation of residual starch in the colon, offering a therapeutic advantage over current AGIs such as acarbose. Additionally, this study revealed that quercetin and rutin did not exhibit cytotoxic effects on Caco‐2 cells for a short‐term period, highlighting their safety profile and supporting their application that could be used as a dietary supplement. Nonetheless, further in vitro studies, such as testing the compounds in an INFOGEST 2.0 simulated gastrointestinal system and metagenomic studies to assess their impact on microbial communities, are needed to validate these compounds as AGI leads. The synergistic effects of acarbose with the flavonoids (quercetin and rutin) should be investigated further.

## Author Contributions

**Itumeleng T. Baloyi:** conceptualization, investigation, methodology, validation, software, formal analysis, data curation, project administration, visualization, writing – original draft. **Ali H. Rabbad:** software, formal analysis, visualization, validation, methodology, data curation, investigation, writing – review and editing. **Ntombenhle H. Gama:** methodology, validation, visualization, writing – review and editing, software, formal analysis, data curation, resources, investigation. **Samkelo Malgas:** conceptualization, investigation, methodology, validation, funding acquisition, writing – review and editing, visualization, supervision, data curation, resources, software, formal analysis, project administration.

## Funding

This research received funding from the National Research Foundation (NRF) of South Africa through the Competitive Support for Unrated Researchers grant (grant number 138084).

## Ethics Statement

The authors have nothing to report.

## Conflicts of Interest

The authors declare no conflicts of interest.

## Supporting information


**Data S1:** fsn371499‐sup‐0001‐Supinfo.zip.

## Data Availability

The data that support the findings of this study are available in the Supporting Information of this article.
