# Supplementary material for: α‐Glucosidase Inhibitory Potential of Citrus reticulata Peel‐Derived Flavonoids—A Prelude for the Management of Type 2 Diabetes
Source: Food Sci Nutr. 2026 Feb 1;14(2):e71499. doi: 10.1002/fsn3.71499 (PMC12862097; doi:10.1002/fsn3.71499)
Supplement: Supplementary file 1 — Data S1: fsn371499‐sup‐0001‐Supinfo.zip. [file FSN3-14-e71499-s001.zip › fsn371499-sup-0002-FigureS1-S6-TableS1-S2@Supplementary material_6695486_Revised.pdf]

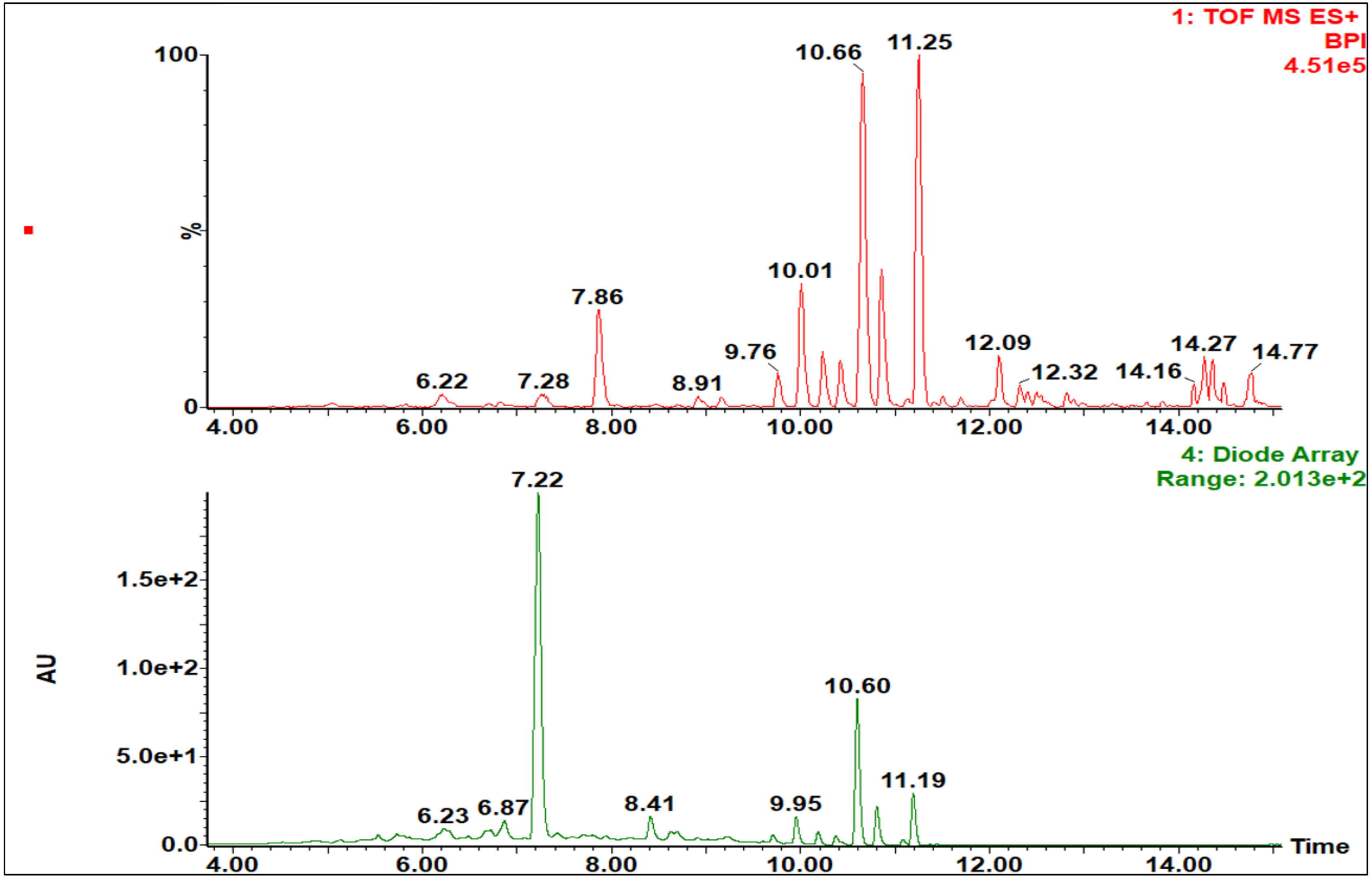

Figure S1: UPLC-chromatograms of the *Citrus reticulata* extract flavonoids detected in the ESI+ mode displayed as a BPI chromatogram, PDA-UV-max.

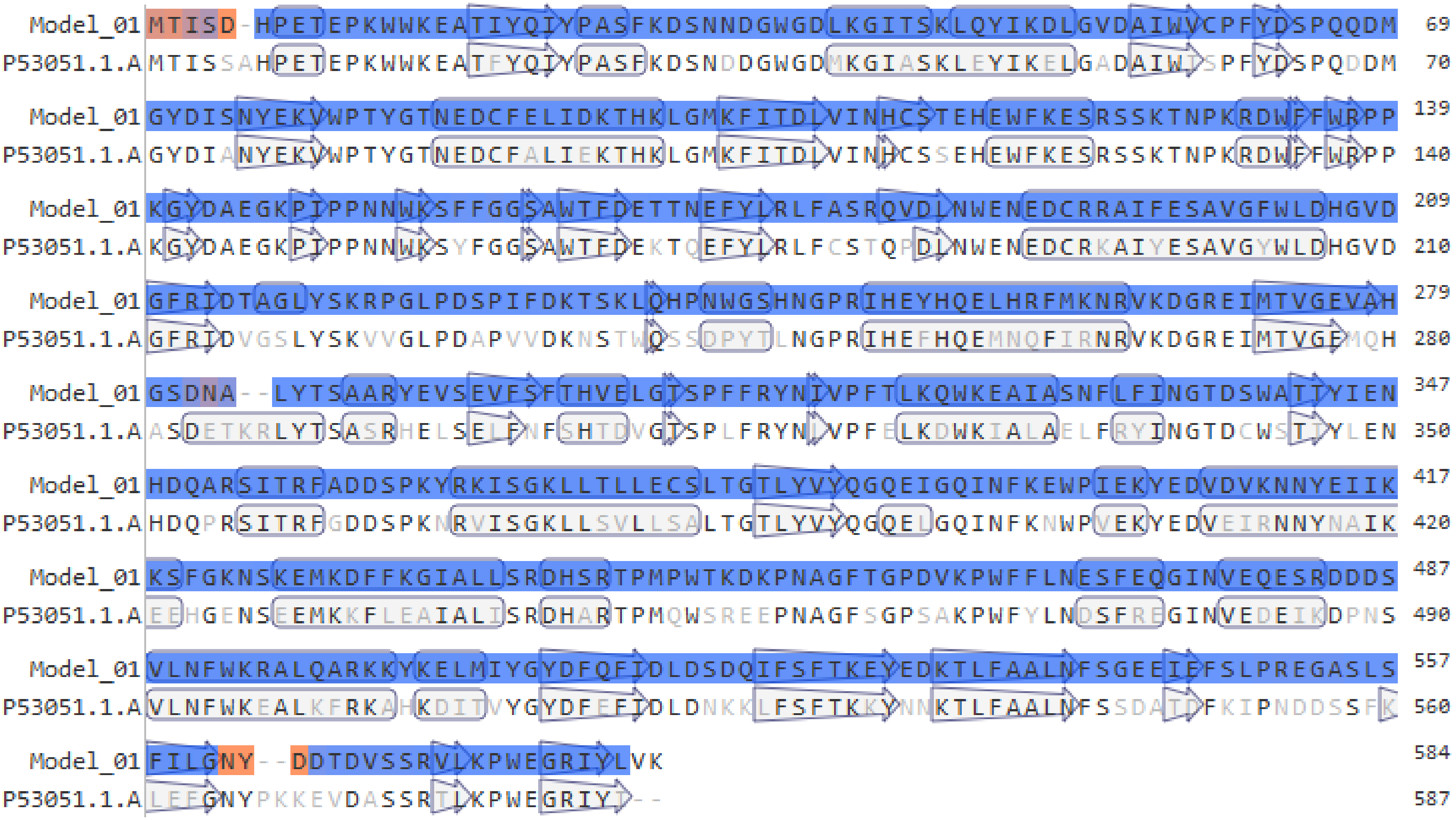

Figure S2. Sequence alignment of the α-glucosidase (MAL32) from *Saccharomyces cerevisiae* (model_01) with the model template, *Saccharomyces cerevisiae* oligo-1,6-glucosidase IMA1 (P53051.1.A). The residues that differ are displayed in a faded style, while similar residues are emphasised. The α-glucosidase sequence from S. cerevisiae is marked in blue, and gaps in the alignment are represented by hyphens (-).

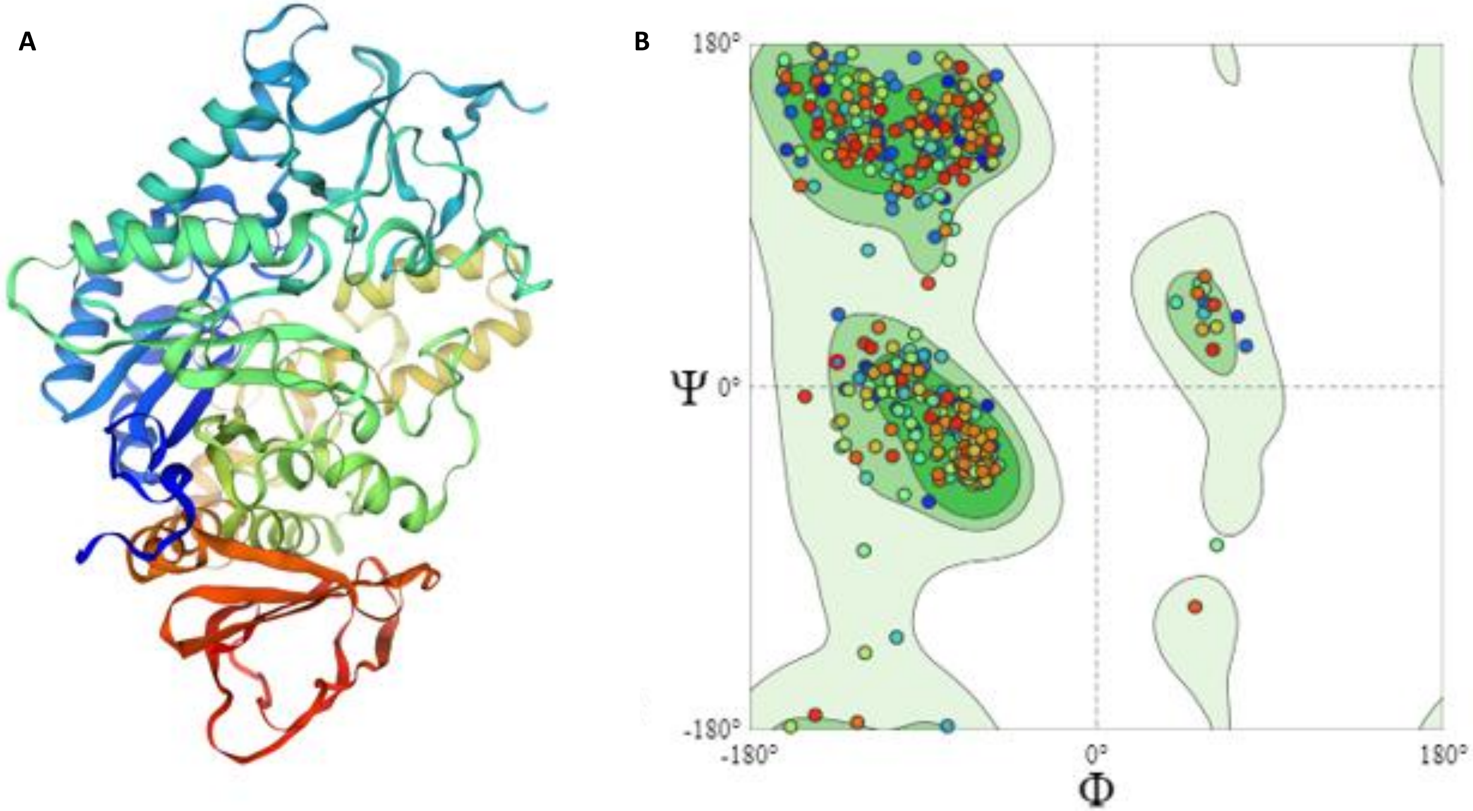

Figure S3. Results from homology modelling demonstrate the (A) 3D structure of α-glucosidase, along with the corresponding (B) Ramachandran plot, indicating the residues located in the favoured and unfavoured regions.

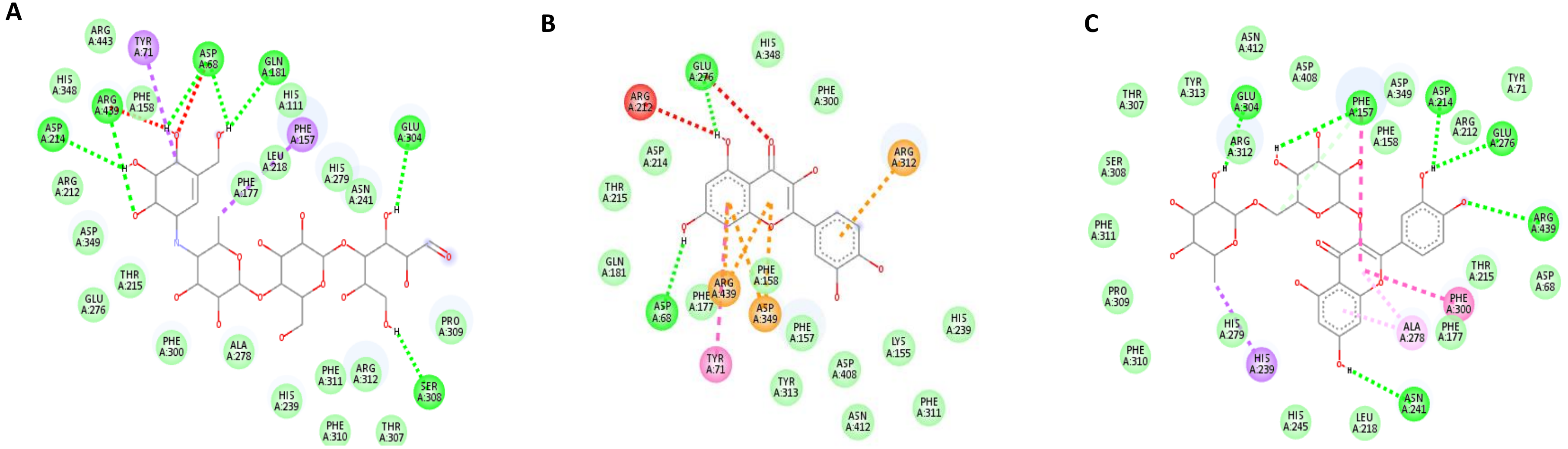

Figure S4. The interaction of (A) acarbose, (B) quercetin, and (C) rutin with the amino acid residues in the active site pocket of α-glucosidase is depicted. Dark green and red lines indicate hydrogen bonds (HB) with the specified protein residues, while all other lines represent Van der Waals interactions (VdW).

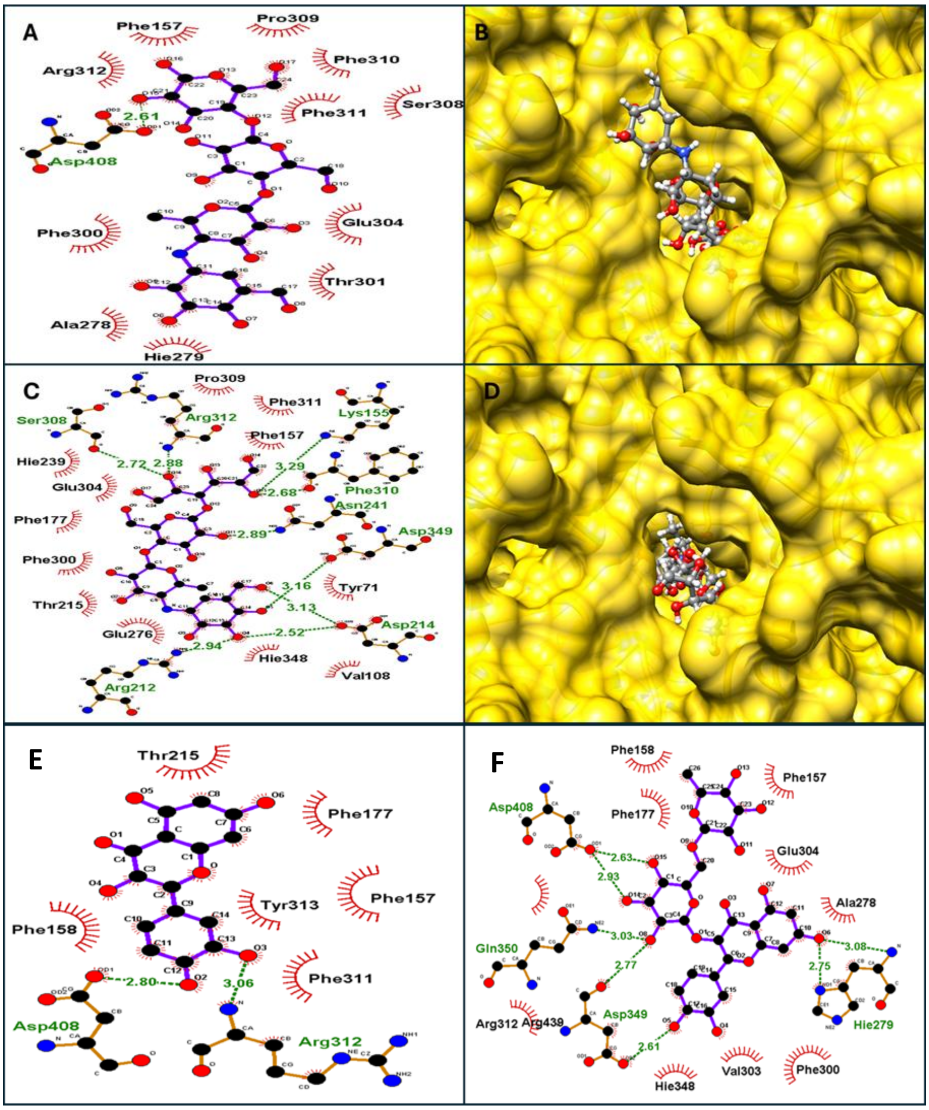

Figure S5. This study presents a comparison of the 2D ligand-protein interaction plots between acarbose and α-glucosidase. (A) illustrates the interactions of acarbose with α-glucosidase, while (B) depicts the binding pose of acarbose as reported in our previous research (Tshiyoyo et al., 2025). In contrast, (C) shows the interactions of acarbose with α-glucosidase as observed in this study and (D) represents the binding pose of acarbose identified in the current research. Network interactions within the α-glucosidase ligand-protein were observed after 100 ns derived from MD simulations. The figure depicts the spatial configuration and types of interactions between the surrounding protein residues and ligands: (E) quercetin and (F) rutin.

Table S1: Total binding energy and conformational dynamics between selected flavonoids and α-glucosidase over 100 ns.

**Compound**	**ΔG_{bind} (kcal/mol)**	**Mean of RMSD (Å)**	**Mean of RMSF (Å)**	**Mean of RoG (Å)**	**Mean of SASA (Å2)**
Apo	-	2.54397	1.26552	24.41912	21710.40
Acarbose	-69.7249	2.1779	1.37601	24.50986	21108.39
Quercetin	-29.1342	2.5086	1.20663	24.57086	21009.19
Rutin	-51.6102	1.66542	1.11694	24.39161	21537.21

Table S2: Average particle size and polydispersity index of starch hydrolysates produced by the amylolytic enzyme cocktail in the absence or presence of inhibitors. Values are represented as means ±SEM (n =3).

**Sample (hydrolysate)**	**Average particle size (mm)**	**Polydispersity (PDI)**
Starch only	156.24±23.34	0.436±0.05
No inhibitor	5.33±0.36	0.298±0.04
Acarbose	140.05±10.09	0.437±0.03
Quercetin	7.13±1.39	0.115±0.07
Rutin	12.57±5.60	0.391±0.11

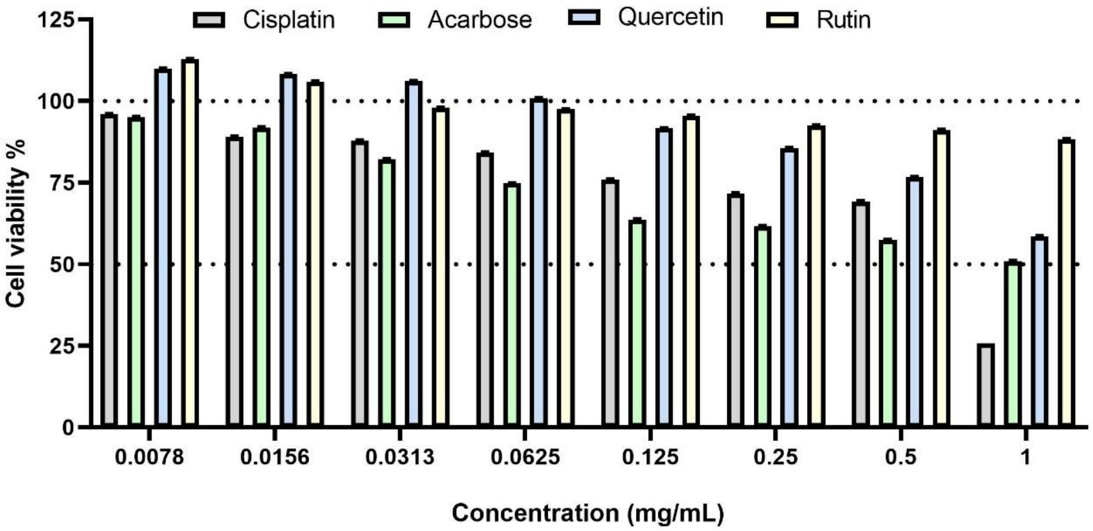

Figure S6. Caco-2 cell viability in the presence of cisplatin, acarbose, quercetin and rutin using MTT assay. Values are represented as means ±SEM (n = 3).
