# Supplementary material for: α‐Glucosidase Inhibitory Potential of Citrus reticulata Peel‐Derived Flavonoids—A Prelude for the Management of Type 2 Diabetes
Source: Food Sci Nutr. 2026 Feb 1;14(2):e71499. doi: 10.1002/fsn3.71499 (PMC12862097; doi:10.1002/fsn3.71499)
Supplement: Supplementary file 1 — Data S1: fsn371499‐sup‐0001‐Supinfo.zip. [file FSN3-14-e71499-s001.zip › fsn371499-sup-0001-FigureS1-S6-TableS1-S2@Supplementary material_21JULY25_JFB.docx]

**Figure section**


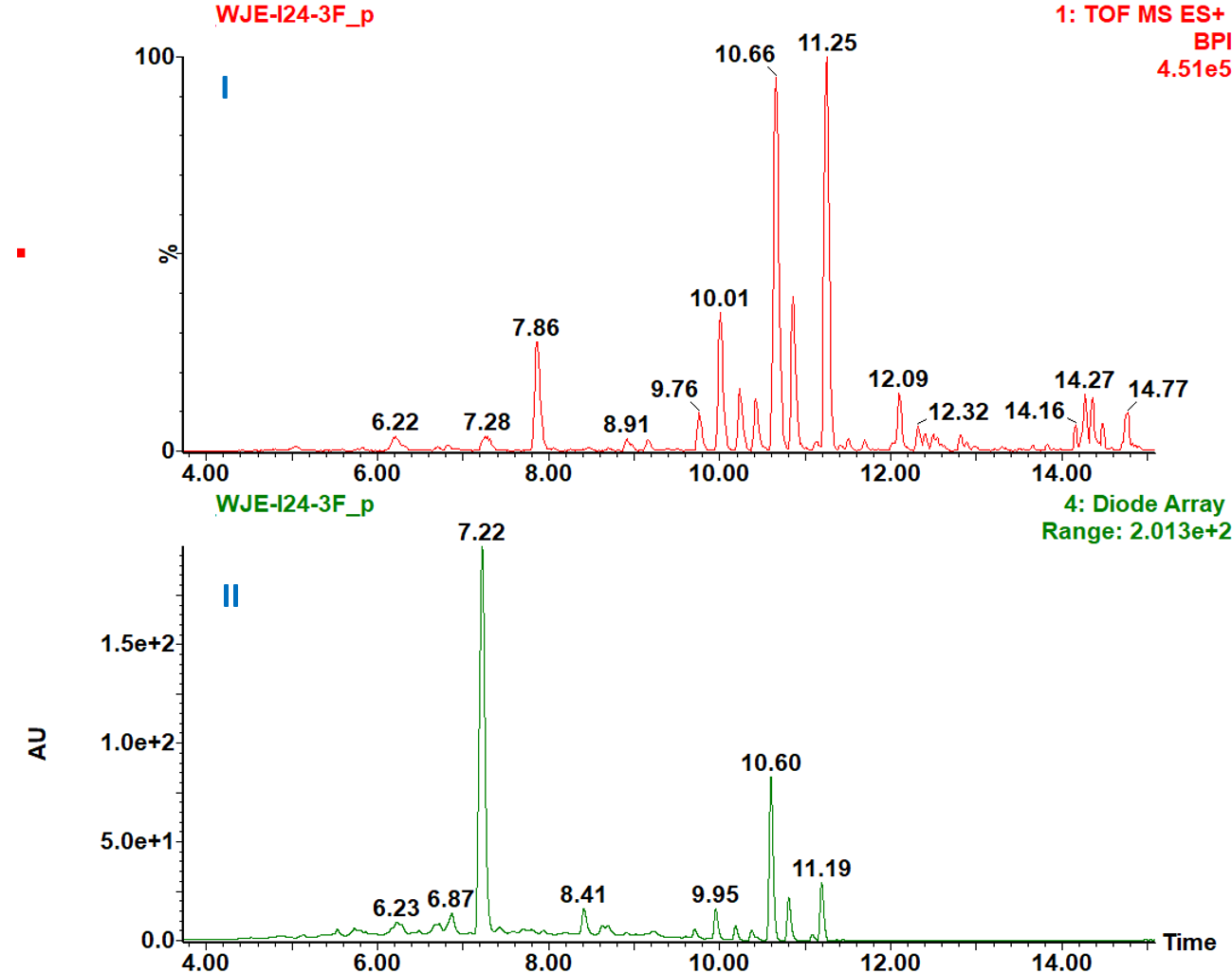


Figure S1: UPLC-chromatograms of *Citrus reticulata* detected in the ESI+ mode displayed as a BPI chromatogram, PDA-UV-max.


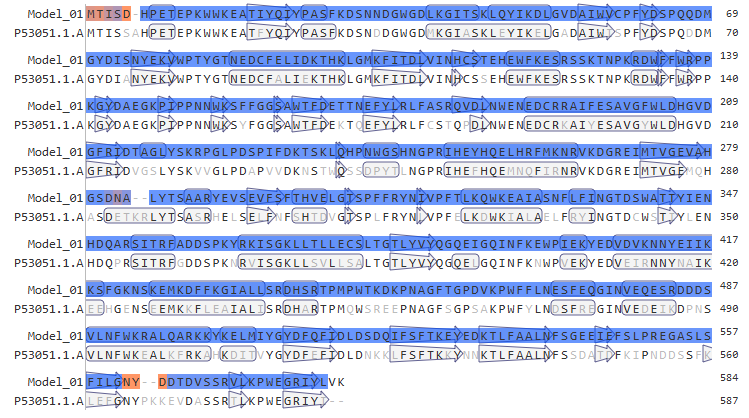


Figure S2. Sequence alignment of the α-glucosidase gene product from *Saccharomyces cerevisiae* (model_01) with the model template, *Saccharomyces cerevisiae* oligo-1,6-glucosidase IMA1 (P53051.1.A). The residues that differ are displayed in a faded style, while similar residues are emphasised. The α-glucosidase sequence from S. cerevisiae is marked in blue, and gaps in the alignment are represented by hyphens (-).


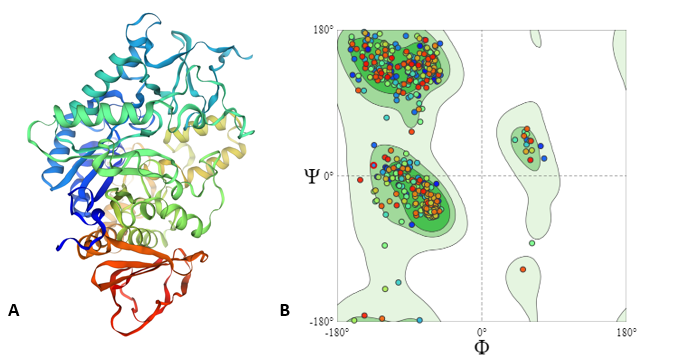


**A**

**B**

Figure S3. Results from homology modelling demonstrate the (A) 3D structure of α-glucosidase, along with the corresponding (B) Ramachandran plot, indicating the residues located in the favoured and unfavoured regions.


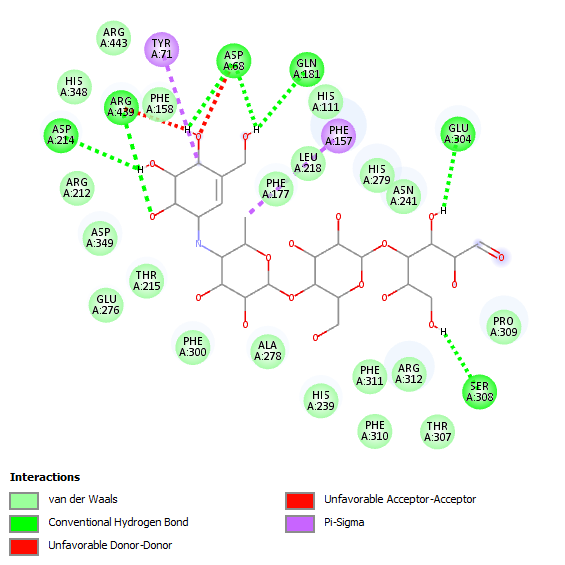

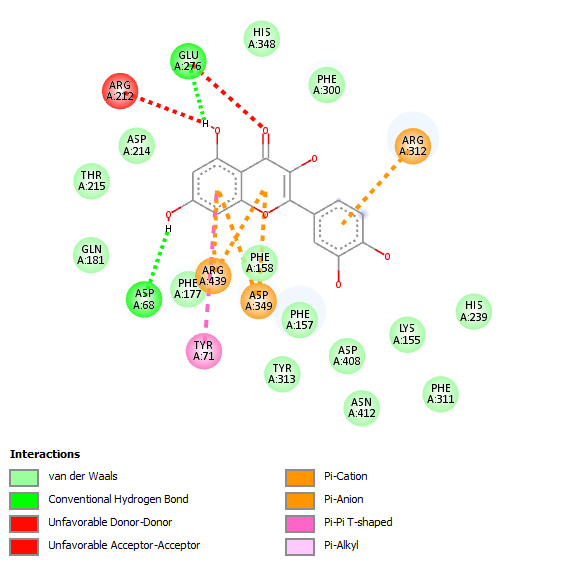

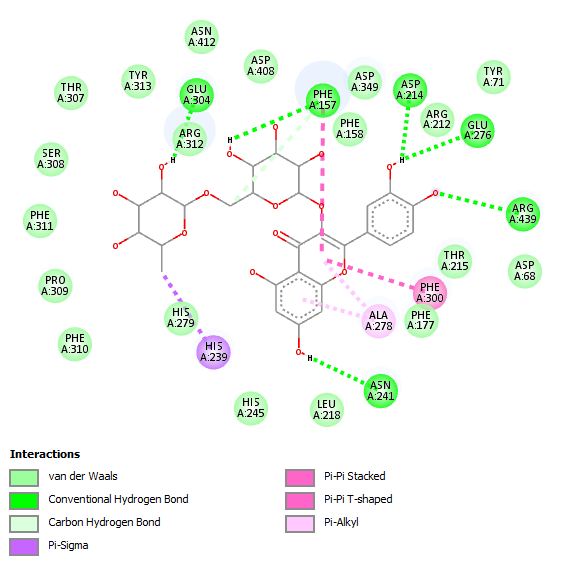


**A**

**B**

**C**

Figure S4. The interaction of (A) acarbose, (B) quercetin, and (C) rutin with the amino acid residues in the active site pocket of α-glucosidase is depicted. Dark green and red lines indicate hydrogen bonds (HB) with the specified protein residues, while all other lines represent Van der Waals interactions (VdW).


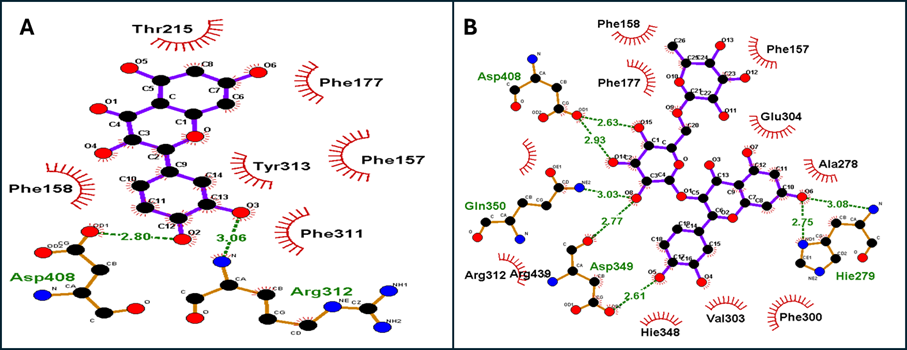


**E**

**F**


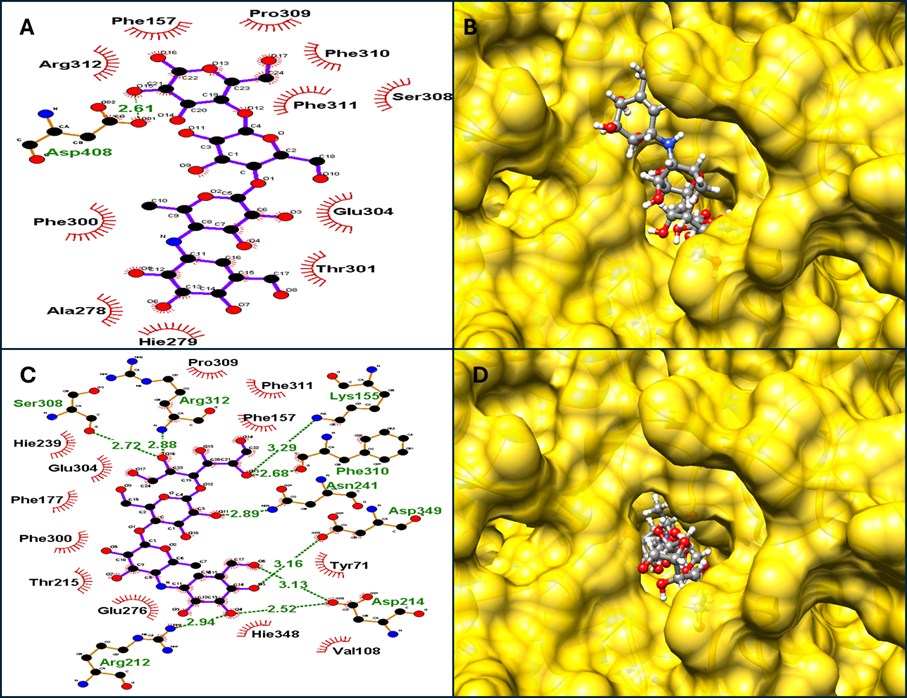


Figure S5. This study presents a comparison of the 2D ligand-protein interaction plots between acarbose and α-glucosidase. (A) illustrates the interactions of acarbose with α-glucosidase, while (B) depicts the binding pose of acarbose as reported in our previous research (Tshiyoyo et al., 2025). In contrast, (C) shows the interactions of acarbose with α-glucosidase as observed in this study and (D) represents the binding pose of acarbose identified in the current research.
Network interactions within the α-glucosidase ligand-protein were observed after 100 ns derived from MD simulations. The figure depicts the spatial configuration and types of interactions between the surrounding protein residues and ligands: (E) quercetin and (F) rutin.


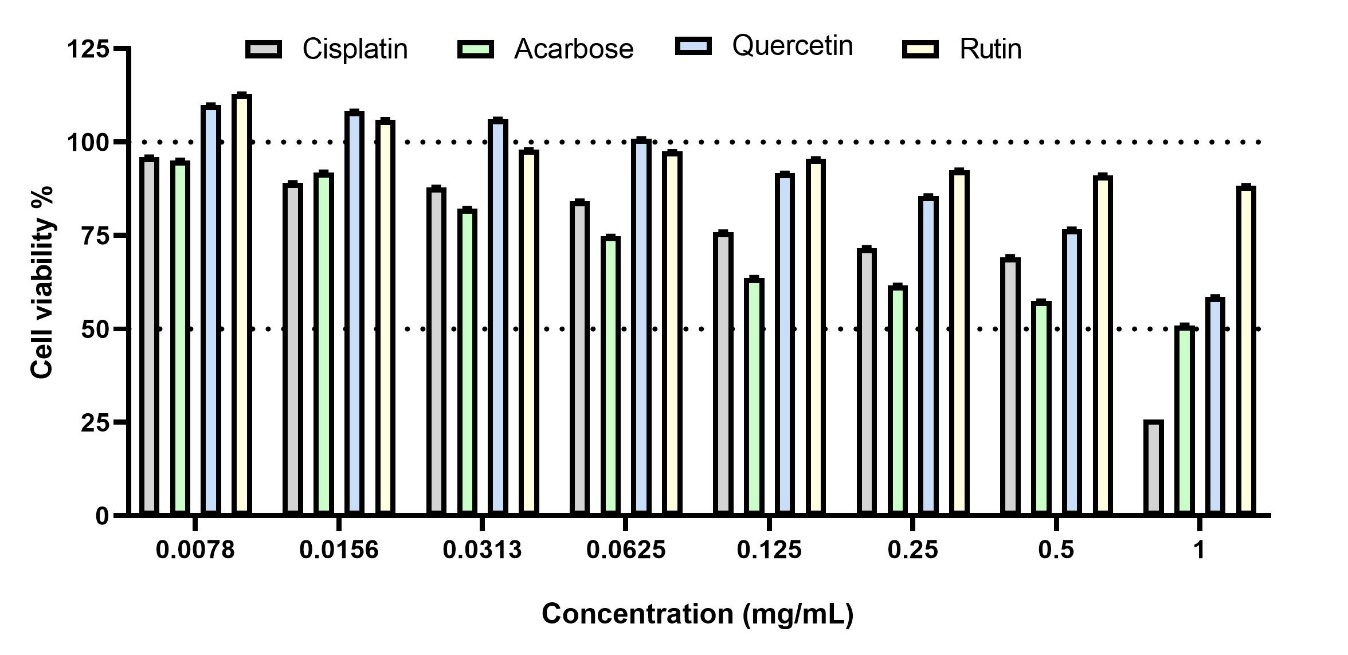


Figure S6: Caco-2 cell viability in the presence of cisplatin, acarbose, quercetin and rutin using MTT assay. Values are represented as means ±SEM (n = 3).

**Table Section**

Table S1: Total binding energy and conformational dynamics between selected flavonoids and α-glucosidase over 100 ns.

| **Compound** | **ΔG_bind_**  **(kcal/mol)** | **Mean of**  **RMSD**  **(Å)** | **Mean of**  **RMSF**  **(Å)** | **Mean of**  **RoG (Å)** | **Mean of SASA**  **(Å2)** |
| --- | --- | --- | --- | --- | --- |
| Apo | - | 2.54397 | 1.26552 | 24.41912 | 21710.40 |
| Acarbose | -69.7249 | 2.1779 | 1.37601 | 24.50986 | 21108.39 |
| Quercetin | -29.1342 | 2.5086 | 1.20663 | 24.57086 | 21009.19 |
| Rutin | -51.6102 | 1.66542 | 1.11694 | 24.39161 | 21537.21 |

Table S2: Average particle size and polydispersity index of starch hydrolysates produced by the amylolytic enzyme cocktail in the absence or presence of inhibitors. Values are represented as means ±SEM (n =3).

| **Sample (hydrolysate)** | **Average particle size (mm)** | **Polydispersity (PDI)** |
| --- | --- | --- |
| Starch only | 156.24±23.34 | 0.436±0.05 |
| No inhibitor | 5.33±0.36 | 0.298±0.04 |
| Acarbose | 140.05±10.09 | 0.437±0.03 |
| Quercetin | 7.13±1.39 | 0.115±0.07 |
| Rutin | 12.57±5.60 | 0.391±0.11 |
